# Expression of estrogen receptor, progesterone receptor, and Ki67 in normal breast tissue in relation to subsequent risk of breast cancer

**DOI:** 10.1038/npjbcancer.2016.32

**Published:** 2016-10-26

**Authors:** Hannah Oh, A Heather Eliassen, Molin Wang, Stephanie A Smith-Warner, Andrew H Beck, Stuart J Schnitt, Laura C Collins, James L Connolly, Laleh Montaser-Kouhsari, Kornelia Polyak, Rulla M Tamimi

**Affiliations:** 1 Department of Epidemiology, Harvard T.H. Chan School of Public Health, Boston, MA, USA; 2 Channing Division of Network Medicine, Brigham and Women’s Hospital and Harvard Medical School, Boston, MA, USA; 3 Department of Nutrition, Harvard T.H. Chan School of Public Health, Boston, MA, USA; 4 Department of Biostatistics, Harvard T.H. Chan School of Public Health, Boston, MA, USA; 5 Department of Pathology, Beth Israel Deaconess Medical Center and Harvard Medical School, Boston, MA, USA; 6 Department of Medical Oncology, Dana-Farber Cancer Institute, Boston, MA, USA; 7 Department of Medicine, Brigham and Women’s Hospital and Harvard Medical School, Boston, MA, USA

## Abstract

Although expression of estrogen receptor (ER), progesterone receptor (PR), and cell proliferation marker Ki67 serve as predictive and prognostic factors in breast cancers, little is known about their roles in normal breast tissue. Here in a nested case–control study within the Nurses’ Health Studies (90 cases, 297 controls), we evaluated their expression levels in normal breast epithelium in relation to subsequent breast cancer risk among women with benign breast disease. Tissue microarrays were constructed using cores obtained from benign biopsies containing normal terminal duct lobular units and immunohistochemical stained for these markers. We found PR and Ki67 expression was non-significantly but positively associated with subsequent breast cancer risk, whereas ER expression was non-significantly inversely associated. After stratifying by lesion subtype, Ki67 was significantly associated with higher risk among women with proliferative lesions with atypical hyperplasia. However, given the small sample size, further studies are required to confirm these results.

Development of breast neoplasia involves hormones such as estrogens and progesterone that regulate cell proliferation and apoptosis. The tissue-specific responsiveness to these hormones is partially regulated by the tissue expression of receptors that bind them.^1^ Ki67 is a cell proliferation marker, as it is present only during active phases of the cell cycle.^2^ Although expression of estrogen receptor α (ER), progesterone receptor (PR), and Ki67 serve as predictive and prognostic factors in breast cancer, little is known about their roles in normal breast tissue. In a nested case–control study within the Nurses’ Health Study (NHS) and NHSII cohorts, we examined the associations of ER, PR, and Ki67 expression levels in normal breast epithelium with subsequent breast cancer risk among women with a previous diagnosis of benign breast disease (BBD).

Briefly, controls were matched to cases on age, calendar year of BBD, and time since biopsy. Archived formalin-fixed paraffin-embedded biopsy blocks were collected from pathology departments for cases and controls. Tissue microarrays (TMAs) were constructed by obtaining 0.6-mm cores of benign lesions and adjacent normal terminal duct lobular units (TDLUs).^3^ A 5-μm section from each TMA block was immunohistochemically stained with each antibody (ERα: clone SP1, Neomarkers, CA, USA; PR: clone PgR 636, Dako Corporation, CA, USA; Ki67: clone SP6, Vector Laboratories, CA, USA). Immunostaining results were interpreted using an automated computational image analysis system (Definiens Tissue Studio software, Munich, Germany); scoring algorithms are shown in Supplementary Figures 1–3. The automated scores were moderately correlated with manually scored data on select TMAs that we scored using both methods (Spearman *r*=0.40–0.48). For each woman, we estimated the mean percentage of stain-positive cells across the cores, by weighting each core by its total cell count. Women were excluded if they had evidence of carcinoma at biopsy or within 6 months of their biopsy, there was not enough tissue on the block for TMA construction, had low cellularity (<100 cells), or had no evaluable marker staining. Details of the methods are described in Supplementary Appendix.

Among 90 breast cancer cases and 297 controls who were included in the analysis, the mean age at BBD biopsy was 45.4 years. The average time between the BBD biopsy and the breast cancer diagnosis was 9.0 years (range: 6 months to 31.3 years). Distributions of population characteristics and marker staining are presented in Supplementary Tables 1 and 2. The median percentages of ER-positive, PR-positive, and Ki67-positive cells in normal TDLUs were 10%, 7%, and 4%, respectively, which were comparable to levels that have been previously reported.^4–6^ Although the expression levels varied slightly by its adjacent lesion type, the differences were not statistically significant (Supplementary Table 3). The intraclass correlation coefficients across cores ranged from 0.26 for Ki67 to 0.45 for ER, suggesting some within-person variability across the cores due to true heterogeneity in tissues^7^ and possibly methodological artifacts. The Spearman correlation for markers in normal TDLUs was modest between ER and PR (*r*=0.35) but minimal between other markers (Supplementary Table 4). When comparing expression levels of normal TDLUs with subsequent breast tumor tissues among cases, the levels were higher in tumor tissue (Supplementary Table 5) as expected^5,6^ and the Spearman correlations were modest for PR (*r*=0.21) and Ki67 (*r*=−0.15) (Supplementary Table 6).

Comparing the highest versus lowest tertiles, ER expression in normal TDLUs was non-significantly but inversely associated with subsequent risk (odds ratio (OR)=0.44, 95% confidence interval (CI)=0.17–1.14, *P *trend=0.68), whereas Ki67 expression was non-significantly but positively associated with subsequent breast cancer risk (OR=1.59, 95% CI=0.80–3.14, *P* trend=0.48; Table 1). PR expression was marginally associated with higher breast cancer risk (OR=2.18, 95% CI=1.04–4.57, *P* trend=0.09); the association was statistically significant when restricted to premenopausal women (OR=3.60, 95% CI=1.26–10.3, *P* trend=0.04). Results were similar after adjustment for BBD subtype. After stratifying by BBD subtype, Ki67 was significantly associated with higher risk among women with atypical hyperplasia (OR=6.80, 95% CI=1.57–29.5, *P* interaction=0.002; Table 2). Results were similar after conducting sensitivity analyses, which included excluding potential outliers, evaluating marker intensity scores, and using alternative cellularity cutpoints for exclusion criteria.

Our finding is consistent with a previous study of women with atypical hyperplasia that reported a fourfold higher breast cancer risk associated with Ki67 expression (⩾2 vs. <2%)^4^ and experimental studies that suggested carcinogenic potentials of PR.^8^ However, we did not observe a positive association with ER despite the supporting evidence.^9,10^ This study has limitations including potential measurement error in markers (for example, lack of data on menstrual phase, moderate correlations between automated and manual scores), which would bias results towards the null.

These findings contribute to our understanding of breast cancer biology and provide the basis for future studies investigating these markers as potential targets for risk assessment among women undergoing biopsies. However, given the small sample size and potential measurement error in markers, further studies are required to confirm these results.

## Figures and Tables

**Table 1:** Odds ratios and 95% confidence intervals of developing subsequent breast cancer according to tertiles of ER, PR, and Ki67 expression in normal breast tissue in the Nurses’ Health Study and the Nurses’ Health Study II

	*Case*	*Control*	*Model 1*^a^ *OR (95% CI)*	*Model 2*^b^ *OR (95% CI)*
*Percentage of ER-positive cells (N=175)*				
<6.9%	17	41	1.0 (Ref)	1.0 (Ref)
6.9–14.6%	15	44	0.69 (0.29, 1.66)	0.71 (0.29, 1.70)
⩾14.7%	10	48	0.44 (0.17, 1.14)	0.45 (0.17, 1.17)
Per 1% increase			0.99 (0.96, 1.03)	0.99 (0.96, 1.03)
*P* trend			0.68	0.67
				
*Percentage of PR-positive cells (N=238)*				
<4.0%	16	64	1.0 (Ref)	1.0 (Ref)
4.0–9.8%	17	61	1.28 (0.58, 2.82)	1.27 (0.57, 2.82)
⩾9.9%	26	54	2.18 (1.04, 4.57)	1.94 (0.91, 4.16)
Per 1% increase			1.03 (1.00, 1.07)	1.03 (0.99, 1.07)
*P* trend			0.09	0.18
				
*Percentage of Ki67-positive cells (*N*=285)*				
<2.4%	20	75	1.0 (Ref)	1.0 (Ref)
2.4–6.1%	18	77	0.92 (0.44, 1.93)	0.98 (0.46, 2.08)
⩾6.2%	28	67	1.59 (0.80, 3.14)	1.79 (0.88, 3.62)
Per 1% increase			1.01 (0.98, 1.05)	1.02 (0.98, 1.06)
*P* trend			0.48	0.29

Abbreviations: CI, confidence interval; ER, estrogen receptor; OR, odds ratio; PR, progesterone receptor.

a Model 1 (adjusted for matching factors only): age at diagnosis (continuous, years), calendar year of benign biopsy (<1970, 1970–1980, >1980), time since benign biopsy (continuous, years).

b Model 2: Model 1+type of benign lesion (non-proliferative, proliferative without atypia, proliferative with atypical hyperplasia).

**Table 2:** Odds ratios and 95% confidence intervals of developing subsequent breast cancer for ER, PR, Ki67 expression^a^ in normal breast tissue stratified by benign lesion type in the Nurses’ Health Study and the Nurses’ Health Study II

	*Non-proliferative lesions*	*Proliferative lesions without atypia*	*Proliferative lesions with atypical hyperplasia*
*ER (⩾10% vs. <10%)*			
Case/control	8/35	18/63	16/35
OR (95% CI)^b^	0.93 (0.18, 4.78)	0.64 (0.21, 1.94)	0.42 (0.10, 1.86)
*P* trend	0.84	0.42	0.45
	*P* interaction^c^=0.69		
			
*PR (⩾6.5% vs. <6.5%)*			
Case/control	10/54	26/90	23/35
OR (95% CI)^b^	1.94 (0.48, 7.87)	1.94 (0.79, 4.74)	2.14 (0.60, 7.73)
*P* trend	0.97	0.16	0.37
	*P* interaction^c^=0.11		
			
*Ki67 (⩾4.2% vs. <4.2%)*			
Case/control	10/58	38/132	18/29
OR (95% CI)^b^	4.29 (0.66, 27.8)	0.62 (0.29, 1.33)	6.80 (1.57, 29.5)
*P* trend	0.05	0.62	0.03
	*P* interaction^c^=0.002		

Abbreviations: CI, confidence interval; ER, estrogen receptor; OR, odds ratio; PR, progesterone receptor.

a ER, PR, and Ki67 expression in normal breast tissue was dichotomized around the median (above versus below median percentage of stain-positive cells).

b Adjusted for matching factors only: age at diagnosis (continuous, years), calendar year of benign biopsy (<1970, >1970), time since benign biopsy (continuous, years).

c *P* interaction was estimated using the likelihood ratio test comparing models with and without the interaction terms between marker expression (high versus low) and BBD lesion type (proliferative lesions with atypical hyperplasia, proliferative lesions without atypia, and non-proliferative lesions).
