# Supplementary material for: Expression of estrogen receptor, progesterone receptor, and Ki67 in normal breast tissue in relation to subsequent risk of breast cancer
Source: NPJ Breast Cancer. 2016 Oct 26;2:16032–. doi: 10.1038/npjbcancer.2016.32 (PMC5243126; doi:10.1038/npjbcancer.2016.32)
Supplement: Supplementary Tables [file npjbcancer201632-s1.doc]

**Supplementary Table 1. Age-standardized characteristics of the study population at benign breast biopsy by breast cancer case-control status in the Nurses’ Health Study and the Nurses’ Health Study II**

|  | **Cases**  **(n=90)** | | **Controls**  **(n=297)** |
| --- | --- | --- | --- |
|  | **Median (IQR)** | | |
| **Stained for ER expression in normal breast TDLUs**  Percentage of ER-positive cells* | 8.8 (5.3 – 13.0) | | 10.4 (4.7 – 16.8) |
| **Stained for PR expression in normal breast TDLUs**  Percentage of PR-positive cells* | 8.6 (3.7 – 12.6) | | 5.6 (2.8 – 11.4) |
| **Stained for Ki67 expression in normal breast TDLUs**  Percentage of Ki67-positive cells* | 5.2 (1.8 – 10.0) | | 4.1 (1.5 – 8.0) |
| **Total number of cells in cores of normal TDLUs*** | 815 (321-1440) | | 701 (351-1385) |
|  |  | |  |
|  | **Mean (SD) or Percentage** | | |
| Age, years* | 45.4 (9.5) | 45.4 (8.9) | |
| Type of benign lesion |  |  | |
| - Non-proliferative, % | 18.9 | 30.7 | |
| - Proliferative without atypia, % | 51.8 | 55.0 | |
| - Proliferative with atypical hyperplasia, % | 29.3 | 14.3 | |
| Height, inches | 64.2 (2.2) | 64.7 (2.6) | |
| Average body size at ages 5-10 years^1^ |  |  | |
| - Level 1 (most lean), % | 34.0 | 34.5 | |
| - Level 1.5-2, % | 42.7 | 28.9 | |
| - Level 2.5-3, % | 7.2 | 16.2 | |
| - Level 3.5-4, % | 13.1 | 11.5 | |
| - Level ≥4.5 (most overweight), % | 2.9 | 9.0 | |
| BMI at age 18 years, kg/m^2^ | 20.9 (2.1) | 21.2 (3.0) | |
| BMI at biopsy, kg/m^2^ | 22.5 (6.3) | 23.1 (5.7) | |
| Age at menarche, years | 12.4 (1.2) | 12.6 (1.4) | |
| Parous, % | 91.1 | 93.6 | |
| Parity (among parous women) | 3.4 (1.6) | 3.1 (1.5) | |
| Age at first birth (among parous women) | 25.3 (3.2) | 24.7 (3.3) | |
| Premenopausal, % | 67.4 | 70.6 | |
| Age at menopause (among postmenopausal women) | 47.4 (4.5) | 46.5 (7.1) | |
| Ever oral contraceptive use, % | 60.3 | 43.8 | |
| Ever smokers, % | 64.5 | 48.7 | |
| First-degree family history of breast cancer, % | 25.1 | 17.8 | |
| Cumulative average lifetime alcohol consumption^2^, g/d | 4.3 (5.0) | 4.6 (6.8) | |
| Cumulative average adult physical activity^3^, MET-hr/wk | 12.5 (12.0) | 16.7 (19.2) | |

Values are standardized to the age distribution of the study population. Values of polytomous variables may not sum to 100% due to rounding.

*Value is not age adjusted.

^1^Participants recalled their body size at ages 5 and 10 years using a 9-level pictogram (level 1: most lean; level 9: most overweight). We averaged body size at ages 5 and 10 years to obtain an estimate of childhood body size.

^2^Cumulative average of alcohol consumption starting at age 18 years to the years prior to benign biopsy.

^3^Cumulative average of physical activity practiced during adulthood since enrollment in the cohort (1976 for the NHS and 1989 for the NHSII) to the years prior to benign biopsy. MET-hr/wk of total activity was estimated by multiplying the number of hr/wk of each activity with its corresponding average MET values (strenuous activity = 7 METs, moderate activity = 4.5 METs, walking = 3 METs) and summing the values from all activities.

Abbreviations: IQR=interquartile range, SD=standard deviation, ER=estrogen receptor, PR=progesterone receptor, TDLU=terminal duct lobular unit, BMI=body mass index, kg=kilogram, g=gram, MET-hr/wk=metabolic equivalent of task-hour/week.

**Supplementary table 2. Total number of cells in cores of normal terminal duct lobular unit by characteristics of the study population**

| **Characteristics** | **N** | **Median (IQR)** | **Mean (SD)** | **p-value^1^** |
| --- | --- | --- | --- | --- |
| **Case-control status** |  |  |  |  |
| Case | 90 | 815 (321-1440) | 1078 (1082) |  |
| Control | 297 | 701 (351-1385) | 1020 (925) | 0.65 |
| **Type of benign lesion** |  |  |  |  |
| Non-proliferative | 106 | 632 (305-1514) | 1023 (1048) |  |
| Proliferative without atypia | 212 | 840 (383-1477) | 1092 (960) |  |
| Proliferative with atypical hyperplasia | 69 | 533 (327-1100) | 869 (812) | 0.25 |
| **Menopausal status** |  |  |  |  |
| Premenopausal | 268 | 858 (387-1520) | 1123 (999) |  |
| Postmenopausal | 119 | 592 (271-1099) | 831 (843) | **0.003** |
| **Parity** |  |  |  |  |
| Nulliparous | 29 | 569 (321-1434) | 974 (875) |  |
| Parous | 358 | 740 (351-1409) | 1038 (970) | 0.71 |
| **Oral contraceptive use** |  |  |  |  |
| Never users | 205 | 794 (395-1385) | 1042 (896) |  |
| Ever users**^2^** | 178 | 647 (298-1440) | 1016 (1044) | 0.79 |
| **Smoking status** |  |  |  |  |
| Never smokers | 182 | 680 (321-1374) | 974 (863) |  |
| Former smokers | 126 | 790 (369-1527) | 1144 (1041) |  |
| Current smokers | 75 | 600 (340-1285) | 984 (1058) | 0.28 |
| **First-degree family history of breast cancer** |  |  |  |  |
| Absent | 311 | 636 (321-1370) | 979 (933) |  |
| Present | 76 | 942 (474-1781) | 1256 (1051) | **0.03** |

^1^p-values were estimated using two sample t-tests (for two categories) or ANOVA tests (for more than two categories).

^2^Former and current oral contraceptive users were combined because there were only few women who were currently using oral contraceptives at benign biopsy.

Abbreviations: IQR=interquartile range; SD=standard deviation

**Supplementary table 3**. Percentages of ER-, PR-, and Ki67-positive cells in normal TDLUs by lesion types at benign biopsy

| **Lesion type at benign biopsy** | | **Percentage of**  **ER-positive cells**  **in normal TDLUs** | **Percentage of**  **PR-positive cells in normal TDLUs** | **Percentage of Ki67-positive cells in normal TDLUs** |
| --- | --- | --- | --- | --- |
| **Non-proliferative lesions** | N | 43 | 64 | 68 |
|  | Median | 10.3 | 5.60 | 4.76 |
|  | Interquartile range | 2.63-18.0 | 2.97-11.2 | 1.87-8.98 |
| **Proliferative lesions without atypia** | N | 81 | 116 | 170 |
|  | Median | 9.14 | 5.60 | 4.49 |
|  | Interquartile range | 4.83-15.8 | 2.76-10.7 | 1.80-9.91 |
| **Proliferative lesions with atypical hyperplasia** | N | 51 | 58 | 47 |
|  | Median | 10.8 | 8.92 | 2.57 |
|  | Interquartile range | 6.31-18.8 | 3.07-14.4 | 1.19-5.98 |

Abbreviations: ER=estrogen receptor; PR=progesterone receptor; TDLU=terminal duct lobular unit

**Supplementary table 4. Spearman correlations among tissue markers in normal breast tissue in the Nurses’ Health Study and the Nurses’ Health Study II**

|  | **All women** | | | **Premenopausal women** | | | **Postmenopausal women** | | |
| --- | --- | --- | --- | --- | --- | --- | --- | --- | --- |
|  | **ER** | **PR** | **Ki67** | **ER** | **PR** | **Ki67** | **ER** | **PR** | **Ki67** |
| **ER** | 1.0 | 0.35  (n=150) | -0.03  (n=110) | 1.0 | 0.44  (n=93) | 0.09  (n=75) | 1.0 | 0.32  (n=41) | -0.27  (n=27) |
| **PR** |  | 1.0 | -0.10  (n=156) |  | 1.0 | -0.01  (n=101) |  | 1.0 | -0.29  (n=45) |
| **Ki67** |  |  | 1.0 |  |  | 1.0 |  |  | 1.0 |

Abbreviations: ER=estrogen receptor; PR=progesterone receptor

**Supplementary table 5. Percentages of ER-, PR-, and Ki7-positive cells in cores of normal terminal duct lobular units, benign lesions, and subsequent tumors**

| **Tissue type** | | **Percentage of**  **ER-positive cells** | **Percentage of**  **PR-positive cells** | **Percentage of Ki67-positive cells** |
| --- | --- | --- | --- | --- |
| **Normal TDLUs** | N | 175 | 238 | 285 |
|  | Median | 9.97 | 6.48 | 4.22 |
|  | Interquartile range | 4.66-16.8 | 2.94-11.5 | 1.49-8.53 |
| **Non-proliferative lesions** | N | 22 | 37 | 26 |
|  | Median | 8.81 | 1.57 | 3.85 |
|  | Interquartile range | 2.78-28.3 | 0.61-7.50 | 1.83-5.88 |
| **Proliferative lesions without atypia** | N | 53 | 75 | 57 |
|  | Median | 11.7 | 8.47 | 3.88 |
|  | Interquartile range | 5.56-20.2 | 5.27-14.4 | 1.55-9.76 |
| **Proliferative lesions with atypical hyperplasia** | N | 31 | 37 | 29 |
|  | Median | 13.4 | 11.5 | 1.66 |
|  | Interquartile range | 7.30-27.5 | 4.44-25.5 | 0.78-3.33 |
| **Subsequent tumor tissue (among cases)** | N | 14 | 17 | 19 |
|  | Median | 44.1 | 18.6 | 5.11 |
|  | Interquartile range | 32.3-54.3 | 1.72-47.7 | 2.71-13.7 |

Abbreviations: ER=estrogen receptor; PR=progesterone receptor; TDLU=terminal duct lobular unit

**Supplementary table 6. Spearman correlations among tissue markers in normal breast tissue vs. breast tumor tissue among cases in the Nurses’ Health Study and the Nurses’ Health Study II**

|  | **Breast tumor tissue** | | |
| --- | --- | --- | --- |
| **Normal tissue** | **ER** | **PR** | **Ki67** |
| **ER** | 0.05  (n=14) | -0.18  (n=14) | -0.11  (n=14) |
| **PR** | -0.10  (n=20) | 0.21  (n=17) | 0.11  (n=20) |
| **Ki67** | -0.20  (n=20) | -0.13  (n=17) | -0.15  (n=19) |

Abbreviations: ER=estrogen receptor; PR=progesterone receptor.
