# Supplementary material for: Expression of estrogen receptor, progesterone receptor, and Ki67 in normal breast tissue in relation to subsequent risk of breast cancer
Source: NPJ Breast Cancer. 2016 Oct 26;2:16032–. doi: 10.1038/npjbcancer.2016.32 (PMC5243126; doi:10.1038/npjbcancer.2016.32)
Supplement: Supplementary Appendix [file npjbcancer201632-s2.doc]

**APPENDIX**

**MATERIALS AND METHODS**

**Study design and population**

This study is a case-control study nested within the subcohort of women who reported a diagnosis of biopsy-confirmed BBD in the NHS and NHSII cohorts. The NHS is an ongoing cohort study that began in 1976, including 121,700 female registered nurses aged 30-55 years. The NHSII is an ongoing cohort study that began in 1989 of 116,430 female registered nurses aged 25-42 years. In both cohorts, initial mailed, self-administered questionnaires collected information on participants’ health behaviors, lifestyle factors, reproductive factors, and medical histories. Subsequent biennial follow-up questionnaires were used to assess updated information on a variety of known and suspected risk factors for chronic diseases, as well as newly diagnosed diseases (e.g., BBD, breast cancer) which were then confirmed via medical record review. The questionnaires were assessed using similar methods in two cohorts. Details of this nested case-control study and the BBD assessment have been previously described . Cumulative response rates for both cohorts were >90% and were similar among women regardless of their BBD diagnosis.

Cases were women with biopsy-confirmed BBD who reported a diagnosis of breast cancer following their BBD diagnosis. Cases were diagnosed during 1976-1998 for the NHS and 1989-1999 for the NHSII. When possible, 4 controls were selected for each case, matched on year of birth and year of benign biopsy, among women with biopsy-confirmed BBD who remained free of breast cancer at the time the matching case was diagnosed. Consequently, age and follow-up time were also matched within matched sets. We attempted to obtain BBD pathology records and archived biopsy specimens for all cases and controls from their hospital pathology departments; our ability to obtain biopsy blocks did not significantly differ by case and control status. Because we were not able to obtain biopsy specimens from all selected cases and controls, there were incomplete matched sets in the study (cases with no matched controls and controls with no matched cases). To reduce potential reverse causation due to subclinical tissue change, women were excluded if they had evidence of *in situ* or invasive carcinoma at biopsy or reported a diagnosis of breast cancer within 6 months of their biopsy (n=34).

This investigation was approved by the Institutional Review Board of the Brigham and Women’s Hospital. Completion of the self-administered questionnaire was presumed to imply informed consent.

**Tissue microarray (TMA) construction and laboratory assays**

Two study pathologists (SJS, JLC) independently reviewed the Hematoxylin and eosin (H&E) stained sections from the benign breast biopsy blocks and completed detailed worksheets on the subtype of BBD lesion (i.e., non-proliferative, proliferative without atypia, atypical ductal hyperplasia [ADH], atypical lobular hyperplasia [ALH]) in a blinded manner. After collecting participants’ archived formalin-fixed paraffin-embedded biopsy blocks, the corresponding H&E slides were re-reviewed by a single pathologist (JLC) to identify the target areas of lesions and adjacent normal TDLUs for TMA coring. Women were excluded if there was no breast tissue, or not enough tissue remaining on the block for coring. Six TMA blocks were constructed in the Dana Farber Harvard Cancer Center Tissue Microarray Core Facility, Boston, MA by obtaining 0.6-mm cores from the targeted area (up to 3 cores for normal TDLUs) in each donor block and inserting them into the recipient TMA blocks. We previously evaluated our TMA construction methods and confirmed a high success rate (76%) of capturing normal TDLUs in these TMA blocks .

For each immunohistochemical (IHC) stain, a 5-μm paraffin section was cut from each TMA block and immunostained with its antibodies (ERα: rabbit monoclonal antibody, clone SP1, RM-9101-S, Neomarkers, CA; PR: mouse monoclonal antibody, clone PgR 636, M3569, Dako Corporation, CA; Ki67: rabbit monoclonal antibody, clone SP6, VP-RM04, Vector Laboratories, CA) after deparaffinizing the section in two 5-min changes of xylene and rehydration through graded alcohols to distilled water. Appropriate positive and negative controls were included in all staining runs. (ER refers to ERα throughout the paper)

Immunostaining results were interpreted using an automated computational image analysis system (Definiens Tissue Studio software, Munich, Germany). For each stain, we used the Tissue Studio software to define an intensity and size threshold for nucleus identification and to define an intensity threshold for nuclear stain positivity. The automated analysis software was trained for scoring only the appropriate epithelial regions of the tissue. Representative images visualizing the scoring algorithm for each stain are shown in **Supplementary Figures 1-3**. Based on these criteria, each cell was classified as positive or negative. Based on the intensity of the nuclear stain, positive cells were further categorized into low-positive, medium-positive, or high-positive. For each woman, we estimated the mean percentage of stain-positive cells (at any intensity) across the cores, by weighting each core by its total cell count, and categorized into tertiles. The percentages of Ki67-positive cells scored by an automated computational image analysis system were moderately correlated with those manually scored (in the percentage of stain-positive cells) by a study pathologist (LCC) on one of the TMAs (Spearman r=0.44). The percentages of ER- and PR-positive cells on five TMAs were manually scored in categories (0%, <1%, 1-9.9%, 10-32.9%, 33-66.9%, ≥67% stain-positive cells); correlations with automated scoring data were also moderate (Spearman r=0.40 for ER, 0.48 for PR). Because manually scored data are more subjective, prone to inter-observer variations, and were available only on a subset of the study population, we used automated scoring data in the analysis. However, for ER and PR which we had data on five TMAs, analysis based on manually scored data yielded similar results, but with much wider confidence intervals. In order to ensure enough cells to estimate the percentage of stain-positive cells, we excluded 42 women with less than 100 total cell count (sum of all the cores); less strict cutoff points (<30, <50 cells) were used in sensitivity analyses. A total of 90 cases and 297 controls with at least one core of normal TDLUs and evaluable ER/PR/Ki67 staining were included in our analyses. There was no significant difference in characteristics (e.g., breast cancer risk factors including BMI, age, hormone use) between women who were included in the analysis, women who were excluded from the analysis due to insufficient cells in cores (<100 cells), and women who were excluded from TMA coring due to insufficient normal TDLUs in biopsy specimens.

Because intensity of the stains is influenced by both storage time (age of block) and variability in processing , as well as the magnitude of expression, we considered intensity in the secondary analyses only by calculating a score encompassing the percentage of stain-positive cells weighted by the intensity of each cell (i.e., intensity score = %low-positive + 2* %medium-positive + 3* %high-positive). To assess heterogeneity across the cores, we calculated ICCs by dividing the between-person variances by the sum of the within- and the between-person variances that were estimated using a linear mixed model.

As previously described , we also constructed breast tumor TMAs, which were then stained and scored for ER, PR, and Ki67 expression, using similar methods among subsequent cases.

**Statistical analysis**

To avoid losing data due to incomplete matched sets (tissue was not available for some cases and controls), unconditional logistic regression, adjusting for matching factors (age, calendar year of BBD biopsy, time since biopsy), was performed to estimate ORs and 95% CIs for breast cancer by tertiles of marker expression, assuming no interaction between matching factors. We tested for the validity of this assumption by comparing results after including interaction terms of matching factors in the models; results did not change, thus, unconditional logistic regression models without the interaction terms were used as our final models. BBD subtype (non-proliferative, proliferative without atypia, proliferative with atypical hyperplasia), an important potential confounder, was additionally adjusted for in the multivariate models. Additional adjustment for other potential confounders, including total cell count and menopausal status, did not change the results, thus were not included in the final models. We performed a test for trend by including exposures in the model as continuous variables. Both linear and non-linear models were assessed again after excluding potential outliers selected based on Quantile-Quantile plots (n=5 for ER, n=4 for PR, n=7 for Ki67). Since levels of ER, PR, and Ki67 expression may vary by menopausal status, we restricted our analyses to women who were premenopausal at BBD biopsy (ER: 25 cases and 87 controls, PR: 33 cases and 115 controls, Ki67: 44 cases and 163 controls) in sensitivity analyses. To assess whether the associations varied by BBD subtype, we estimated the associations for marker expression (above vs. below the median) after stratifying the study population by BBD subtype (non-proliferative, proliferative without atypia, proliferative with atypical hyperplasia). Likelihood ratio tests compared models with and without interaction terms to determine significance of the interactions between marker expression and BBD subtype. All statistical tests were two-sided with 5 % type I error. Analyses were conducted with SAS version 9 (SAS Institute).

**REFERENCES:**

1. Collins LC, Baer HJ, Tamimi RM, Connolly JL, Colditz GA, Schnitt SJ. The influence of family history on breast cancer risk in women with biopsy-confirmed benign breast disease: results from the Nurses' Health Study. Cancer. 2006 Sep 15;107(6):1240-7. PubMed PMID: 16902983.

2. Tamimi RM, Colditz GA, Wang Y, Collins LC, Hu R, Rosner B, et al. Expression of IGF1R in normal breast tissue and subsequent risk of breast cancer. Breast cancer research and treatment. 2011 Jul;128(1):243-50. PubMed PMID: 21197570. Pubmed Central PMCID: 3116083.

3. Page DL, Dupont WD, Rogers LW, Rados MS. Atypical hyperplastic lesions of the female breast. A long-term follow-up study. Cancer. 1985 Jun 1;55(11):2698-708. PubMed PMID: 2986821.

4. Collins LC, Wang YH, Connolly JL, Baer HJ, Hu R, Schnitt SJ, et al. Potential Role of Tissue Microarrays for the Study of Biomarker Expression in Benign Breast Disease and Normal Breast Tissue. Appl Immunohisto M M. 2009 Oct;17(5):438-41. PubMed PMID: WOS:000270287100012. English.

5. Atkins D, Reiffen KA, Tegtmeier CL, Winther H, Bonato MS, Storkel S. Immunohistochemical detection of EGFR in paraffin-embedded tumor tissues: variation in staining intensity due to choice of fixative and storage time of tissue sections. The journal of histochemistry and cytochemistry : official journal of the Histochemistry Society. 2004 Jul;52(7):893-901. PubMed PMID: 15208356.

6. Tamimi RM, Baer HJ, Marotti J, Galan M, Galaburda L, Fu Y, et al. Comparison of molecular phenotypes of ductal carcinoma in situ and invasive breast cancer. Breast cancer research : BCR. 2008;10(4):R67. PubMed PMID: 18681955. Pubmed Central PMCID: 2575540.

7. Collins LC, Marotti JD, Baer HJ, Tamimi RM. Comparison of estrogen receptor results from pathology reports with results from central laboratory testing. Journal of the National Cancer Institute. 2008 Feb 6;100(3):218-21. PubMed PMID: 18230800. Pubmed Central PMCID: 4014130.

8. Collins LC, Botero ML, Schnitt SJ. Bimodal frequency distribution of estrogen receptor immunohistochemical staining results in breast cancer: an analysis of 825 cases. American journal of clinical pathology. 2005 Jan;123(1):16-20. PubMed PMID: 15762275.
