# Supplementary figures and images for: Expression of estrogen receptor, progesterone receptor, and Ki67 in normal breast tissue in relation to subsequent risk of breast cancer
Source: NPJ Breast Cancer. 2016 Oct 26;2:16032–. doi: 10.1038/npjbcancer.2016.32 (PMC5243126; doi:10.1038/npjbcancer.2016.32)

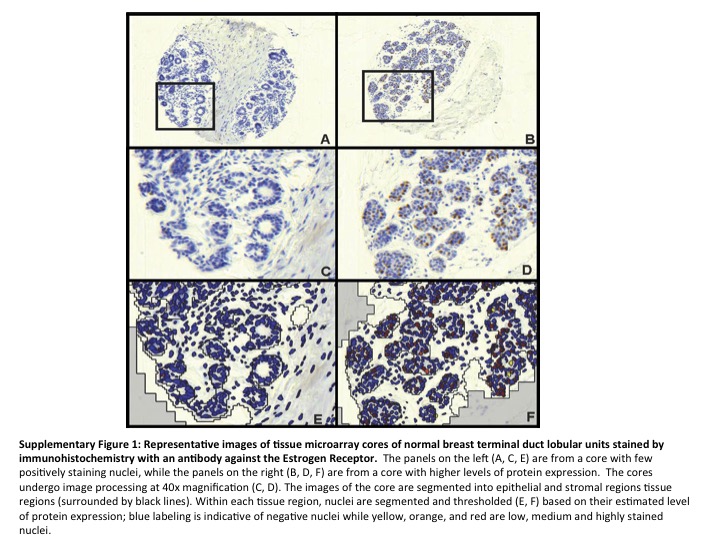

Supplement: Supplementary Figure 1 [file npjbcancer201632-s3.jpg]

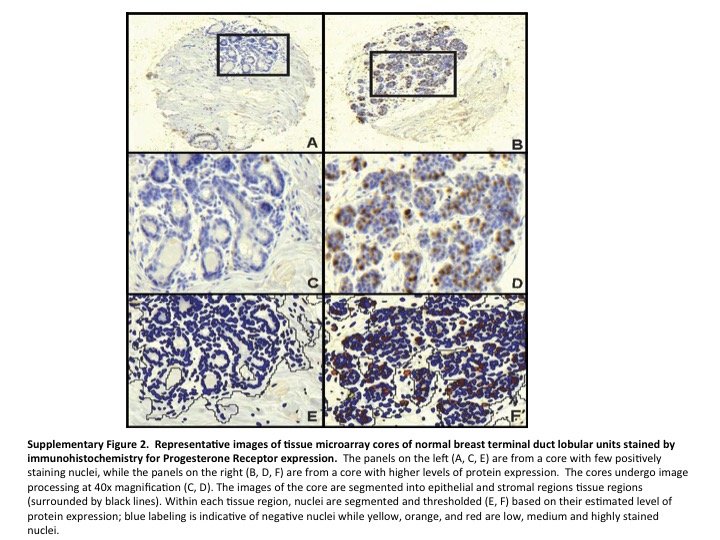

Supplement: Supplementary Figure 2 [file npjbcancer201632-s4.jpg]

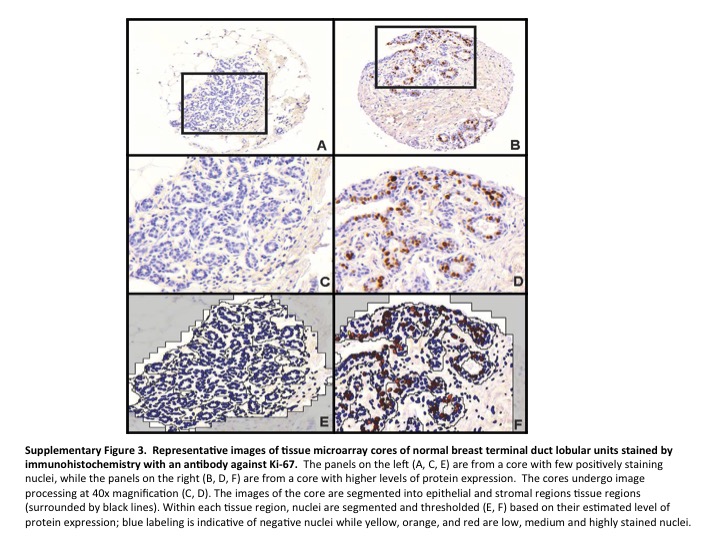

Supplement: Supplementary Figure 3 [file npjbcancer201632-s5.jpg]
